# Lutetium [^177^Lu]-DOTA-TATE in gastroenteropancreatic-neuroendocrine tumours: rationale, design and baseline characteristics of the Italian prospective observational (REAL-LU) study

**DOI:** 10.1007/s00259-024-06725-7

**Published:** 2024-05-22

**Authors:** Secondo Lastoria, Marcello Rodari, Maddalena Sansovini, Sergio Baldari, Antonio D’Agostini, Anna Rita Cervino, Angelina Filice, Matteo Salgarello, Germano Perotti, Alberto Nieri, Davide Campana, Riccardo Emanuele Pellerito, Elena Pomposelli, Valeria Gaudieri, Giovanni Storto, Chiara Maria Grana, Alberto Signore, Giuseppe Boni, Francesco Dondi, Gabriele Simontacchi, Ettore Seregni

**Affiliations:** 1 Division of Nuclear Medicine, IRCCS Istituto Nazionale Tumori, “Fondazione Senatore Giovanni Pascale”, Naples, Italy; 2 Nuclear Medicine Unit, IRCCS–Humanitas Clinical and Research Hospital, Rozzano, MI Italy; 3 Nuclear Medicine Unit, IRCCS Istituto Romagnolo per lo Studio dei Tumori (IRST), “Dino Amadori”, Meldola, Italy; 4 Department of Biomedical and Dental Sciences and Morpho-Functional Imaging, University of Messina, Messina, Italy; 5 U.O.C. Medicina Nucleare-Ospedale “S.M. Goretti”, Latina, Italy; 6 Nuclear Medicine Unit, Veneto Institute of Oncology IOV–IRCCS, Padua, Italy; 7 Nuclear Medicine Unit, AUSL-IRCCS of Reggio Emilia, Reggio Emilia, Italy; 8 Nuclear Medicine Department, Sacrocuore-Don Calabria Hospital, Negrar di Valpolicella, Italy; 9 Nuclear Medicine Unit, Fondazione Policlinico Universitario A. Gemelli-IRCCS, Rome, Italy; 10 Nuclear Medicine Unit, Onco-Hematological Department, University Hospital of Ferrara, Ferrara, Italy; 11 Oncology Unit, IRCCS Azienda Ospedaliero-Universitaria di Bologna, Bologna, Italy; 12 Nuclear Medicine Unit, Ordine Mauriziano Hospital, Turin, Italy; 13 SC Medicina Nucleare, Azienda Ospedaliera-Universitaria SS Antonio e Biagio e Cesare Arrigo, Alessandria, Italy; 14 Department of Advanced Biomedical Sciences, University ‘‘Federico II’’ of Naples, Naples, Italy; 15 IRCCS CROB Referral Cancer Center of Basilicata, Rionero in Vulture (Pz), Italy; 16 Radiometabolic Therapy, Nuclear Medicine, IRCCS European Institute of Oncology, Milan, Italy; 17 Nuclear Medicine Unit, Department of Medical-Surgical Sciences and Translational Medicine, University Hospital Sant’Andrea, “Sapienza” University of Rome, Rome, Italy; 18 Nuclear Medicine Therapy Unit, Azienda Ospedaliero-Universitaria Pisana, Pisa, Italy; 19 Nuclear Medicine, ASST Spedali Civili di Brescia, Brescia, Italy; 20 SODc Radiotherapy, Department of Oncology, Azienda Ospedaliero-Universitaria Careggi, Florence, Italy; 21 Nuclear Medicine Unit, IRCCS–Fondazione Istituto Nazionale dei Tumori, Milan, Italy

**Keywords:** [^177^Lu]Lu-DOTA-TATE, Gastroenteropancreatic-neuroendocrine tumours, Italy, Peptide receptor radionuclide therapy, REAL-LU, Real-world

## Abstract

**Purpose:**

Gastroenteropancreatic -neuroendocrine tumours (GEP-NETs) are commonly treated with surgical resection or long-term therapies for tumour growth control. Lutetium [^177^Lu]-DOTA-TATE was approved for the treatment of GEP-NETs after the phase III NETTER 1trial demonstrated improved progression free survival, objective response rates and health-related quality of life (HRQoL) compared to high-dose somatostatin analogues.

No real-world data exist on prescribing habits and clinically significant endpoints for [^177^Lu]Lu-DOTA-TATE treatment in Italy. REAL-LU is a multicentre, long-term observational study in patients with unresectable/metastatic GEP-NETs progressing on standard therapies in Italian clinical practice. A pre-specified interim analysis was performed at the end of the enrolment period, data from which are described herein.

**Methods:**

Overall duration of REAL-LU will be approximately 48 months, with 12- and 36-month recruitment and follow-up periods, respectively. The primary objective is to evaluate [^177^Lu]Lu-DOTA-TATE effectiveness in terms of progression-free survival. Secondary objectives include safety, impact on HRQoL, and identification of prognostic factors. This pre-specified interim analysis describes patient profiles, at the end of enrollment, of those prescribed [^177^Lu]Lu-DOTA-TATE for GEP-NETs in Italy.

**Results:**

Among 161 evaluable patients, mean age was 64.7 ± 10.3 years at study entry, 83.8% presented with no clinical signs of disease at physical examination, and most had minor disease symptoms. All patients had metastatic disease, most commonly in the liver (83.9%) with a median of two metastatic sites. In 90.7% of patients, the disease was stage IV, and 68.3% had ≥ 1 target lesion. [^177^Lu]Lu-DOTA-TATE was prescribed mainly as second-line therapy (61.6%) and following surgery (58.4%). HRQoL assessments revealed high levels of functioning and low levels of symptoms at baseline; 50.0% of patients were symptom-free at study entry.

**Conclusion:**

The characteristics of patients who received [177Lu]Lu-DOTA-TATE in Italy are similar to those of the GEP-NET population of NETTER 1 with trial but with a higher proportion of patients with a grade 2 (71%). With regard to the tumor grade profile, our study cohort appears to be closer to that of NETTER-2 study population which included patients with G2 or G3 advanced GEP-NETs (i.e. Ki-67 ≥ 10% and ≤ 55%). Further analysis of effectiveness and safety can be anticipated as REAL-LU data mature.

Study Registration: ClinicalTrials.gov, NCT04727723; Study Registration Date: 25 January, 2021; https://clinicaltrials.gov/study/NCT04727723?cond=NCT04727723&rank=1

**Supplementary Information:**

The online version contains supplementary material available at 10.1007/s00259-024-06725-7.

## Introduction

Neuroendocrine tumours (NETs) constitute a heterogeneous group of rare malignant neoplasms originating from the neuroendocrine cell system [1–3]. The presentation, prognosis, molecular features, clinical behaviour and location of NETs are variable, although the gastrointestinal tract is the most common location [4]. Such tumours can be referred to as gastroenteropancreatic-NETs (GEP-NETs). There is evidence that the incidence and prevalence of NETs are increasing [1, 5–7], which can be explained, at least in part, by improved detection of early-stage disease and stage migration [5].

Surgical resection of the primary tumour and metastatic lesions remains the primary treatment with curative intent and is associated with the best long-term outcomes [8, 9]. However, only a minority of patients are cured by surgery because many patients with GEP-NETs are diagnosed at an advanced tumour stage (when surgical intervention is not an option) and are offered long-term systemic treatment for symptomatic relief and tumour growth control [1, 9].

In the last decade, tumour-targeted peptide receptor radionuclide therapy (PRRT) became available for use alone or in combination with cytoreductive procedures (i.e. surgery or liver-directed procedures) [9]. [^177^Lu]Lu-DOTA-TATE, ^177^Lu-DOTA^0^-Tyr^3^-Octreotate, ^177^Lu-DOTATOC, ^177^Lu DOTA-octreotate, ^177^Lu oxodotreotide or Lutathera® (Advanced Accelerator Applications) are all radioligands binding to somatostatin receptors (SSTRs) and consist of an SSA (octreotate) coupled to the metal-ion chelating moiety, DOTA, and radiolabelled with ^177^Lu [10]. [^177^Lu]Lu-DOTA-TATE binds with high-affinity to SSTRs, emitting low- to intermediate-energy beta-particles with a tissue penetration range of up to 2 mm, which directs most of the radiation dose to the target tumour with slight loss to the surrounding tissues [10].

[^177^Lu]Lu-DOTA-TATE was granted approval in Europe on 26 September 2017 for treating unresectable or metastatic, progressive, well-differentiated (G1 and G2) GEP-NETs [11] and in the United States on 29 January 2018 for the treatment of SSTR-positive GEP-NETs [12]. These approvals were based on the results of the randomised controlled phase III NETTER-1 trial, which demonstrated that treatment with [^177^Lu]Lu-DOTA-TATE resulted in markedly longer progression-free survival (PFS) and a significantly higher response rate than high-dose octreotide long-acting release in patients with advanced midgut NETs [13]. Approval was further supported by data from the phase I/II ERASMUS study [14, 15].

PRRT with [^177^Lu]Lu-DOTA-TATE has improved objective response rates (ORR), PFS, and overall survival (OS) in patients with GEP-NETs, compared with the current standard of care and is associated with limited and primarily reversible side effects [14–16]. Additionally, [^177^Lu]Lu-DOTA-TATE has been shown to improve health-related quality of life (HRQoL), including global health status, physical and role functioning, and clinically relevant disease-related symptoms [17].

Although [^177^Lu]Lu-DOTA-TATE was beneficial in a well-defined patient population in the NETTER-1 trial, no real-world data are available on the prescribing habits and clinically significant endpoints for [^177^Lu]Lu-DOTA-TATE in a clinical practice setting in Italy. Furthermore, only limited data on [^177^Lu]Lu-DOTA-TATE are available in Europe. The prospective, multicentre observational REAL-LU (pRospective obsErvationAL study to assess the effectiveness and outcomes associated with LUtathera; ClinicalTrials.gov, NCT04727723) study was designed to capture real-world data on the baseline characteristics of patients with unresectable/metastatic, SSTR-expressing, well-differentiated (Grade 1 or 2) GEP-NETs prescribed [^177^Lu]Lu-DOTA-TATE in a clinical setting in Italy, and to gain insights into the effectiveness and safety outcomes in these patients. Moreover, the study aimed to assess the impact of [^177^Lu]Lu-DOTA-TATE treatment on HRQoL and identify possible prognostic factors related to clinical effectiveness outcomes. The REAL-LU study differs in design both from NETTER-1 and the most recent NETTER 2 trial, which were open-label randomised phase III trials comparing [^177^Lu]Lu-DOTA-TATE and high-dose long-acting octreotide in selected GEP-NET populations of [13, 18]. REAL-LU is an observational study evaluating the real-world effectiveness and outcomes of [^177^Lu]Lu-DOTA-TATE in a potentially more heterogeneous patient population, as seen during standard clinical practice. A planned, descriptive interim analysis was performed at the end of the enrolment period for the REAL-LU study. Herein, we report on the patient profile of those prescribed [^177^Lu]Lu-DOTA-TATE in a real-world Italian setting. Response and outcome data will be reported when data are sufficiently mature.

## Methods

### Study design and patient population

REAL-LU (NCT04727723) is a national, multicentre, long-term observational study of adult patients diagnosed with unresectable or metastatic, progressive, well-differentiated (G1 and G2), SSTR-positive GEP-NETs who have been selected for treatment with [^177^Lu]Lu-DOTA-TATE in routine clinical practice in Italy. The study is being conducted at 21 sites and plans to enrol 150 patients. Data will be collected during routine clinical visits and examinations, starting from the date of the patient’s informed consent and ending when the last enrolled patient has completed 36 months of assessments (unless early termination is required). The total study duration will be approximately 48 months, with 12 months of recruitment and 36 months of follow-up after the last patient has been enrolled.

**Fig. 1:**
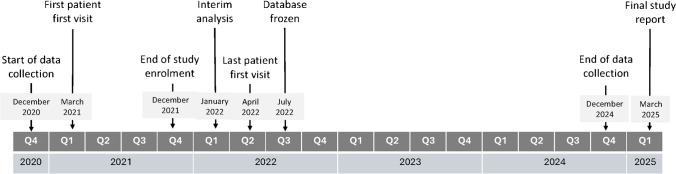
Key study milestones and dates

Key study milestones are shown in Fig. 1.

This paper provides a pre-specified descriptive analysis performed at the end of the enrolment period (12 months), to gain insights into the baseline characteristics of patients treated with [^177^Lu]Lu-DOTA-TATE at authorised centres in Italy.

### Study ethics

The study was sponsored by Advanced Accelerator Applications and was designed and conducted ethically in accordance with the Declaration of Helsinki, Guidelines for Good Pharmacoepidemiology Practices [19], Strengthening the Reporting of Observational Studies in Epidemiology guidelines [20] and following all relevant requirements of local laws in Italy.

The investigational review board or local ethics committee at each participating site reviewed and approved the study protocol, and the rights, safety, and well-being of all participants were further protected by the regulations of Advanced Accelerator Applications, Italy, and by the strict monitoring and reporting requirements stipulated by Agenzia Italiana del Farmaco (AIFA).

Protocol and report writing, project management, statistics and data analysis, and regulatory and monitoring activities of the study were carried out by the contract research organisation (CRO), OPIS S.r.l., Desio, Italy.

### Study objectives and endpoints

The complete study objectives and endpoints of REAL-LU are presented in Table 1. The primary objective is to evaluate [^177^Lu]Lu-DOTA-TATE effectiveness in terms of PFS. ORR, duration of response (DoR), clinical benefit rate (CBR), duration of clinical benefit, and time to progression (TTP) are secondary objectives. The safety of [^177^Lu]Lu-DOTA-TATE and the impact of treatment on HRQoL will also be assessed, as well as the time to deterioration (TTD) in selected HRQoL items/scales.

**Table 1:** REAL-LU study objectives and endpoints

Objectives	Endpoints
Primary	
Evaluate the effectiveness of [^177^Lu]Lu-DOTA-TATE treatment in terms of PFS	PFS, defined as the time (months) from treatment initiation to the date of first objective tumour progression according to RECIST 1.1 criteria or death due to any cause, whichever comes first
Secondary	
Evaluate the effectiveness of treatment in terms of ORR	ORR, defined as the proportion of treated patients who achieve a best overall response of PR or CR according to RECIST 1.1
Evaluate the effectiveness of treatment in terms of DoR, for patients who achieve a best response of PR or better	DoR, defined as the time (months) from the date when response criteria are first met until the date of a progression event
Evaluate the effectiveness of treatment in terms of CBR	CBR, defined as the proportion of treated patients who achieve a best overall response of SD, PR or CR
Evaluate the effectiveness of treatment in terms of duration of clinical benefit, for those patients who achieve a best response of SD or better	Duration of clinical benefit, defined as the time (months) from the date when criteria for clinical benefit are first met until the date of a progression event
Evaluate the effectiveness of treatment in terms of TTP	TTP, defined as the time (months) from treatment initiation to the date of first objective tumour progression, according to RECIST 1.1
Assess the impact of treatment on HRQoL	Changes from baseline in HRQoL will be assessed using the EORTC QoL questionnaires (EORTC QLQ-C30 and EORTC QLQ-G.I.NET-21) assessed during routine clinical visits until the end of study, disease progression, or death, collected and evaluated in relation to objective tumour response, KPS scores and other parameters of clinical relevance
Evaluate the effectiveness of treatment in terms of TTD in selected HRQoL items/scales	TTD, defined as the time (months) from treatment initiation to the date of first deterioration of ≥ 10 points in selected HRQoL domain score compared to the baseline score for the same domain
Evaluate treatment safety	Incidence and severity of AEs, seriousness and relationship to treatment, and action taken; any-cause deaths; changes in laboratory parameters, vital signs, physical examination, ECG results and KPS scores
Describe baseline characteristics of patients selected for treatment	Baseline characteristics of patients prescribed [^177^Lu]Lu-DOTA-TATE (medical and disease history, prior treatments for NETs, baseline and demographic characteristics)
Explore the correlation of possible prognostic factors with clinical effectiveness outcomes	Potential prognostic factors (e.g. SSTR expression levels determined by Octreoscan® scintigraphy or ^68^ Ga PET/CT according to clinical practice, standardised uptake value of [^18^F]FDG PET/CT [if performed], levels of the biomarkers collected in clinical routine, stage of disease at the time of first diagnosis, KPS score at baseline)
Describe radiation emission levels at 1 m distance of patients treated with [^177^Lu]Lu-DOTA-TATE	Radiation emission levels at 1 m distance of treated patients at hospital discharge and collected according to the local SmPC, the “*Scheda di Monitoraggio* AIFA” and as per clinical practice
Describe dosimetry data after [^177^Lu]Lu-DOTA-TATE administration	Number of patients undergoing dosimetry, dosimetry method used and radiation-absorbed doses to tumour and normal organs after administration
Evaluate health resource usage	Number of days of hospitalisation for [^177^Lu]Lu-DOTA-TATE treatment; frequency and duration of hospitalisation; extent of usage of concomitant medications for AE treatment; changes in use of concomitant medications for symptoms management; information about the patient’s diagnosis-related group

*AEs* Adverse events; *AIFA* Agenzia Italiana del Farmaco; *CBR* Clinical benefit rate; *CR* Complete response; *CT* Computed tomography; *DoR* Duration of response; *ECG* Electrocardiogram; *EORTC* European Organization for Research and Treatment of Cancer; *FDG* Fluorodeoxyglucose; *HRQoL* Health-related quality of life; *KPS* Karnofsky performance status; *NETs* Neuroendocrine tumours; *ORR* Objective response rate; *PET* Positron emission tomography; *PFS* Progression-free survival; *PR* Partial response; *QoL* Quality of life; *RECIST* Response Evaluation Criteria in Solid Tumours; *SD* Stable disease; *SmPC* Summary of Product Characteristics; *SSTR* Somatostatin receptor; *TTD* Time to deterioration; *TTP* Time to progression

Additional secondary objectives are to describe the baseline characteristics of patients selected for [^177^Lu]Lu-DOTA-TATE treatment (reported herein) and explore the correlation of possible prognostic factors with clinical effectiveness outcomes.

### Inclusion and exclusion criteria

A complete list of inclusion and exclusion criteria are presented in Table 2. In all participants, the decision to initiate treatment with [^177^Lu]Lu-DOTA-TATE had to occur before patients were enrolled in REAL-LU; treatment must not have been initiated for the purpose of participating in the study.

**Table 2:** Key inclusion and exclusion criteria in the REAL-LU study

Inclusion criteria
Aged ≥ 18 years
Confirmed diagnosis of unresectable or metastatic, progressive, well-differentiated (G1 and G2), SSTR-positive GEP-NET
Naïve to treatment with [^177^Lu]Lu-DOTA-TATE
Able to provide written informed consent prior to any data collection
Exclusion criteria
Participated in an investigational study within 30 days preceding enrolment or within 5 half-lives of the investigational product, whichever is longer
Did not meet the eligibility, prescribing, contraindications and administration criteria states in the local SmPC [11]

*GEP-NET* Gastroenteropancreatic-neuroendocrine tumour; *SmPC* Summary of Product Characteristics; *SSTR* Somatostatin receptor

### Study measures

Patient baseline clinical characteristics and demographics are collected and recorded in an electronic case report form (eCRF), including complete medical history, primary diagnosis, and disease status, the radiographic imaging technique used for tumour assessment, information from surgery/biopsy specimens (including chromogranin-A (CgA) and synaptophysin expression if available), SSTR expression levels, prior treatments, and concomitant medications.

Disease status includes disease stage according to the European Neuroendocrine Tumour Society (ENETS) Tumour Node Metastasis (TNM) classification, tumour grade (according to Ki-67 index or/and mitotic count), presence and site(s) of metastases, Karnofsky Performance Status (KPS) score, and any relevant clinical signs and symptoms.

All prescription and over-the-counter medications taken from enrolment until the end of treatment are to be recorded, as are all procedures and non-drug therapies (e.g. physical therapy and blood transfusions). The medication history will include information relating to any chemotherapy, hormonal therapy, immunotherapy, radiation therapy, or surgery that the patient has previously received for NETs and related symptoms, previous treatment with SSAs, and any anticancer treatments administered after disease progression.

All adverse events (AEs) or patient deaths, regardless of their relationship to treatment, will be recorded until the end of the study. AEs observed in the 30 days following [^177^Lu]Lu-DOTA-TATE treatment will only be recorded if suspected of being associated with [^177^Lu]Lu-DOTA-TATE. All dosing details, including any treatment modifications, discontinuations, or interruptions, will also be recorded with reasons. Patients discontinuing treatment before the end of the treatment period will be monitored for survival.

Laboratory assessments, electrocardiograms (ECGs), physical assessment, vital signs, and KPS will be monitored per clinical practice and the summary of product characteristics (SmPC) and any clinically relevant laboratory abnormality will be recorded.

HRQoL will be assessed using European Organization for Research and Treatment of Cancer (EORTC) quality of life questionnaires, specifically EORTC QLQ-C30 [21] and EORTC QLQ-G.I.NET-21 [22]. The EORTC QLQ-C30 incorporates multi-item scales, such as functional, symptom, and global health status/QoL scales. The EORTC QLQ-G.I.NET-21 is specific for patients with NETs and comprises 21 questions assessing disease symptoms, treatment side effects, body image, disease-related worries, social functioning, communication, and sexuality. The G.I.NET-21 domains include an endocrine scale (i.e. flushing and sweats), gastrointestinal scale (i.e. bloating and flatulence), treatment scale, social functioning scale, disease-related worries scale, muscle/bone pain, sexual function, information/communication function, and body image scales. Higher G.I.NET-21 scores correspond to increased symptom severity.

All data collected in the eCRF are loaded into the study database by the investigator and/or study coordinator using a fully-validated electronic data capture (EDC) software system; the investigator/study coordinator is also responsible for quality control. The designated CRO is responsible for reviewing the data for completeness, accuracy, and to ensure database quality processes.

### Treatment procedure

[^177^Lu]Lu-DOTA-TATE is to be administered according to the recommended treatment regimen in adults consisting of four equally divided doses totalling 29.6 GBq (or 800 millicuries [mCi]). Treatment duration is in accordance with the local SmPC [11], the AIFA monitoring form and clinical practice. The recommended interval between each administration is 8 weeks, which can be extended to 16 weeks in case of dose-modifying toxicity. Follow-up visits and imaging will be conducted according to routine clinical practice and following guidelines for the diagnosis, treatment, and follow-up of GEP-NETs [9]. Acceptable imaging methods are described in Supplementary Methods, Online Resource 1.

### Statistical analysis

Statistical analyses will have descriptive and exploratory purposes; therefore, the alpha level will not be adjusted for primary and secondary outcome variables. No formal sample size calculation was performed for the overall target sample size of 150 patients. The estimated precision of PFS probability is based on the pre-specified sample size, assuming an expected censoring probability of 0.35 and an expected 50.0% of patients surviving at 28.5 months (similar to the median PFS observed in the phase I/II Erasmus MC study) [15].

The analysis sets comprise the enrolled population (patients who provided written informed consent and were suitable for [^177^Lu]Lu-DOTA-TATE treatment), the safety population (patients who received ≥ 1 dose of study treatment), and the evaluable population (patients who received ≥ 1 dose of study treatment and have ≥ 1 post-baseline tumour assessment).

Any discrepancies or missing values will be recorded by the EDC system, and imputations, if deemed appropriate, will be reported. Statistical significance will be considered as a two-sided alpha level of 0.05. No inferential analysis will be performed. Analysis of pooled and summarised data, including continuous and categorical data, will be performed by the CRO. All statistical analyses will be performed using SAS® version 9.4 or later (SAS Institute, Inc, Cary, NC, USA).

## Results

The interim analysis was performed in the enrolled population at the end of the enrolment period. The cut-off date was extended from 2 March 2022 to 28 April 2022, to ensure that the planned sample size of 150 patients was achieved.

### Baseline demographics

A total of 164 patients were enrolled from 21 authorised centres in Italy (Supplementary Table S1, Online Resource 1), and 161 patients were included in the evaluable population (3 patients did not receive [^177^Lu]Lu-DOTA-TATE and were excluded). At study entry, the mean age was 64.7 ± 10.3 years and all but one patient were Caucasian. Of the 74 women enrolled, 64 (86.5%) were menopausal, 8 (10.8%) were of childbearing potential, and 2 (2.7%) were sterile. Patient characteristics are summarised in Table 3.

**Table 3:** Baseline patient demographics and clinical characteristics of the REAL-LU evaluable population

Characteristic	*N* = 161
Age at study entry, years	
Mean ± SD	64.7 ± 10.3
Median (range)	66.0 (33.0–82.0)
Gender, *n* (%)	
Female	74 (46.0)
Male	87 (54.0)
KPS score, n (%)^a^	130 (80.8)
Mean ± SD	92.7 ± 7.6
KPS score 60	1 (0.8)
KPS score 70	2 (1.5)
KPS score 80	12 (9.2)
KPS score 90	61 (46.9)
KPS score 100	54 (41.5)
Mean ± SD age at first diagnosis of GEP-NET, years	59.9 ± 10.7
Time from diagnosis to start of [^177^Lu]Lu-DOTA-TATE therapy, years	
Mean ± SD	4.8 ± 4.7
Median (interquartile range)	3.7 (1.3–7.3)
Primary tumour site at diagnosis,^b^ *n* (%)	
Ileum	70 (43.5)
Pancreas	58 (36.0)
Other^c^	37 (22.9)
Metastatic disease at study entry, *n* (%)	161 (100.0)
Number of sites, median (range)	2.0 (1.0–5.0)
Site of metastases at study entry, *n* (%)	
Liver	135 (83.9)
Regional lymph nodes	88 (54.7)
Peritoneum	37 (23.0)
Bone	37 (23.0)
Distant lymph nodes	30 (18.6)
Other^d^	28 (17.4)

*GEP-NET* Gastroenteropancreatic-neuroendocrine tumour; *KPS* Karnofsky Performance Status; *SD* Standard deviation

^a^KPS score 60: requires occasional assistance but is able to care for most of his/her personal needs; KPS score 70: cares for self; unable to carry on normal activity or to do active work; KPS score 80: normal activity with effort; some signs or symptoms of disease; KPS score 90: able to carry on normal activity; minor signs or symptoms of disease; KPS score 100: normal no complaints; no evidence of disease

^b^Four patients had more than one primary tumour site, so percentages sum to > 100%

^c^Other includes the stomach, duodenum, jejunum, cecum, ascending colon, sigmoid colon, and rectum, each in < 5.0% of patients

^d^Other includes lung, ileum, mediastinum, pelvis (non-bone), jejunum and pleura, each in < 5.0% of patients

Baseline KPS was assessed in 130 patients; the median score was 90.0 (range, 60.0–100.0), indicating that most patients could carry on with normal activities and had only minor signs or symptoms of disease. One hundred and thirty-five patients (83.8%) appeared ‘normal’ at physical examination.

At the time of this interim analysis, 46 (28.6%) patients had completed treatment, 106 (65.8%) were receiving ongoing treatment, and 9 (5.6%) had discontinued treatment. Reasons for discontinuing treatment included the occurrence of an AE (*n* = 3; one event each of hypoglycaemic coma, respiratory failure, and pulmonary embolism), death (*n* = 2), disease progression (*n* = 2), physician decision due to occurrence of thrombocytopenia (*n* = 1), and patient withdrawal (*n* = 1).

At the end of study enrolment, 154 patients (95.7%) were receiving ongoing treatment, and 7 (4.4%) had discontinued treatment due to death (*n* = 2), disease progression (*n* = 2), patient withdrawal (*n* = 2), or physician decision due to occurrence of thrombocytopenia (*n* = 1). Most patients (90.1%) had ≥ 1 previous or concomitant disease or previous surgery (Supplementary Table S2, Online Resource 1).

### Tumour characteristics

At the time of GEP-NET diagnosis, the most frequent primary tumour sites were the ileum (43.5%) and the pancreas (36.0%; Table 3). Most patients (*n* = 157, 97.5%) had a single tumour site and four (2.5%) had two tumour sites; these were ileum + caecum in one patient, ileum + appendix in one patient, and pancreas + other site in two patients. At study entry, all evaluable patients (*n* = 161) had metastatic disease, most commonly in the liver (83.9%), followed by regional lymph nodes (54.7%), the peritoneum (23.0%), bone (23.0%), and distant lymph nodes (18.6%). Among these patients, the median number of metastatic sites was 2.0 (range 1.0–5.0), with a single metastatic site observed in 29.2% of patients, two in 35.4%, three in 22.4%, four in 8.7%, and five sites in 4.4% of patients.

Tumour grade (according to the Ki-67 index and/or mitotic count) was evaluated in 86 (53.4%) patients at study entry. The Ki-67 index was most frequently between 3.0–20.0% (*n* = 61, 70.9%) and was < 3.0% in 24 (27.9%) patients. The mitotic rate was available in 21/86 patients, with a median value of 2.0 (range, 0–16.0).

Most patients (90.7%) had stage IV disease at study entry, with histopathological grade 1 and 2 disease in 70.9% and 27.9% of patients, respectively. According to the Response Evaluation Criteria in Solid Tumours (RECIST) Criteria, version 1.1, 110 (68.3%) patients at study entry had ≥ 1 target lesion, 89 (55.3%) had ≥ 1 non-target lesion, 42 (26.1%) had ≥ 1 nodal target lesion, and 82 (50.9%) had ≥ 1 non-nodal target lesion.

### Somatostatin receptor expression

SSTR expression was evaluated in 152 (94.4%) patients, predominantly using a ^68^Ga-DOTATOC-positron emission tomography (PET)/computed tomography (CT) scan (96.7%; Table 4). Standardised uptake value (SUV) was performed in 113 (74.3%) patients. The mean maximum SUV was 45.0 ± 36.1 and the median SUV was 29.4 (range, 3.8–167.0). Among 39 (25.6%) patients with a Krenning score evaluation, 29 (74.4%) patients had a Krenning score of 4, and 4 patients each (10.3%) had scores of 2 and 3.

**Table 4:** Somatostatin receptor status and fluorodeoxyglucose parameters at REAL-LU study entry

Parameter	*N* = 161
SSTR expression evaluated at study entry, *n* (%)	152 (94.4)
Type of SSTR imaging system at study entry^a^, *n* (%)	
^68^ Ga-DOTATOC PET/CT scan	147 (96.7)
SRS with OctreoScan	1 (0.7)
Other^b^	4 (2.6)
Type of quantification, *n* (%)	
SUV^a^	113 (74.3)
Krenning score	39 (25.7)
Maximum SUV^c^, mean ± SD	45.0 (36.1)
Krenning score^d^, *n* (%)	
Score 2	4 (10.3)
Score 3	4 (10.3)
Score 4	29 (74.4)
NA	2 (5.1)
FDG PET/CT evaluation, n (%)	50 (31.1)
Positive	22 (44.0)
Negative	28 (56.0)

*CT* Computed tomography; *FDG* Fluorodeoxyglucose; *NA* Not available; *PET* Positron emission tomography; *SD* Standard deviation; *SRS* Somatostatin receptor scintigraphy; *SSTR* Somatostatin receptor; *SUV* Standardised uptake value

Percentages were based on patients with ^a^SSTR expression evaluation; ^b68^Ga-DOTA-TATE PET/CT, ^68^ Ga-DOTANOC PET/CT, or SSTR scintigraphy with Tektrotyd; ^c^SUV and ^d^Krenning score evaluation at study entry

In some participating centres, [^18^F]fluorodeoxyglucose (FDG) PET/CT scans were performed alongside ^68^Ga-DOTATOC-PET/CT scans if a discordance between positive CT lesions and negative ^68^Ga-DOTATOC-PET lesions was observed, or there was rapid disease progression. FDG uptake in known lesions was evaluated in 50 (31.1%) patients and was positive in 22 (44.0%) patients.

The mean time to initiate [^177^Lu]Lu-DOTA-TATE therapy was 4.8 ± 4.7 years after GEP-NET diagnosis; in other words, treatment was initiated at an early disease stage. Notably, [^177^Lu]Lu-DOTA-TATE was prescribed mainly as a second-line therapy after progression following SSA treatment. Ninety-eight (61.6%) patients received second-line [^177^Lu]Lu-DOTA-TATE.

### Prior procedures and treatments for GEP-NETs

Most evaluable patients had undergone a prior procedure for GEP-NETs (*n* = 98, 60.9%); primarily surgery (*n* = 94, 58.4%), followed by locoregional therapies (*n* = 13, 8.1%), radiotherapy (*n* = 1) and other prior procedures (*n* = 2). Among previous surgical procedures, the most frequent were ileectomy (*n* = 28, 29.8%), hepatectomy and lymphadenectomy (*n* = 20, 21.3% each), colectomy and pancreatectomy (*n* = 15, 16.0% each), and pancreaticoduodenectomy, pancreaticosplenectomy, and cancer surgery (*n* = 6, 6.4% each). Prophylactic cholecystectomy was reported in 12.8% of patients; any other surgical procedures for GEP-NETs were reported in < 5 patients each.

All 159 patients (98.8%) who had previously or were currently receiving treatment for GEP-NETs had taken or were taking SSAs. Of the 126 (78.3%) patients receiving ongoing GEP-NET therapies at study entry, all were receiving SSAs, mainly lanreotide acetate (48.4%), followed by octreotide acetate (18.3%) and octreotide or lanreotide (16.7% each). SSA therapy was prescribed as first- or second-line treatment in 30.2% and 6.3% of patients with GEP-NETs of the pancreas, respectively, and in 56.0% and 12.0% of patients with gastrointestinal GEP-NETs, respectively.

In the cohort of 159 patients with available information on prior or ongoing therapies for GEP-NETs at study entry, 61.6% were receiving [^177^Lu]Lu-DOTA-TATE as second-line therapy, 29.6% as third-line, and 8.8% as later-line treatment. Within the subgroup of patients with GEP-NETs of the pancreas, [^177^Lu]Lu-DOTA-TATE was second-, third- and later-line treatment in 54.4%, 29.8% and 15.8%, respectively, and in those with gastrointestinal GEP-NETs the corresponding proportion of patients receiving [^177^Lu]Lu-DOTA-TATE second-, third- or later-line treatment was 67.0%, 28.7% and 4.3%, respectively.

### Health-related quality of life

Baseline HRQoL was assessed in 155 (96.3%) patients and is summarised in Table 5. Briefly, when HRQoL was assessed using the EORTIC QLQ-C30 questionnaire, mean functional, symptom, global health, and single-item scale scores indicated high levels of functioning, low levels of symptoms and high QoL. Notably, median scores were 100 for role, cognitive, and social functioning scales. Symptoms such as fatigue, nausea, and vomiting were absent, and approximately 50.0% of patients were symptom-free at baseline.

**Table 5:** Health-related quality of life parameters at REAL-LU study entry

	Total (*n* = 155)	
EORTC QLQ-C30	Mean (SD)	Median (range)
Functional scales		
Physical	87.0 (15.6)	93.3 (16.7–100.0)
Role	85.7 (22.6)	100.0 (0.0–100.0)
Emotional	76.9 (20.0)	83.3 (0.0–100.0)
Cognitive	88.7 (16.8)	100.0 (16.7–100.0)
Social	86.8 (21.0)	100.0 (0.0–100.0)
Symptom scales		
Fatigue	23.0 (21.0)	22.2 (0.0–100.0)
Nausea and vomiting	5.6 (12.1)	0.0 (0.0–66.7)
Pain	5.6 (12.1)	0.0 (0.0–66.7)
Global health scale	68.1 (21.2)	66.7 (0.0–100.0)
Single-item scales		
Dyspnoea^a^	1.3 (0.5)	1.0 (1.0–3.0)
Sleep disturbance	1.6 (0.7)	1.0 (1.0–4.0)
Appetite loss^a^	1.2 (0.5)	1.0 (1.0–4.0)
Constipation	1.3 (0.6)	1.0 (1.0–4.0)
Diarrhea^a^	1.6 (0.8)	1.0 (1.0–4.0)
Financial difficulties	1.3 (0.6)	1.0 (1.0–4.0)
EORTC QLQ-G.I.NET-21		
Scales		
Endocrine	11.1 (16.6)	0.0 (0.0–77.8)
Gastrointestinal	19.6 (16.1)	20.0 (0.0–73.3)
Treatment^b^	11.3 (16.5)	0.0 (0.0–83.3)
Social function	32.3 (21.3)	33.3 (0.0–100.0)
Disease-related worries^a^	42.3 (24.2)	33.3 (0.0–100.0)
Single-item scales		
Body image^c^	1.3 (0.6)	1.0 (1.0–4.0)
Muscle/bone pain^a^	1.6 (0.8)	1.0 (1.0–4.0)
Information/communication function	1.2 (0.5)	1.0 (1.0–4.0)
Sexual function^d^	1.8 (1.0)	1.0 (1.0–4.0)

*EORTC QLQ-C30* European Organization for Research and Treatment of Cancer quality of life C30 questionnaire; *EORTC QLQ-G.I.NET-21* EORTC neuroendocrine tumour-specific quality of life questionnaire; *SD* Standard deviation

^a^*n* = 154; ^b^*n* = 93; ^c^*n* = 149; ^d^*n* = 114

Evaluation of baseline HRQoL using the NET-specific EORTC QLQ-G.I.NET-21 questionnaire revealed only a small impact on disease symptoms, treatment side effects, body image, disease-related worries, social functioning, communication, and sexuality. Higher mean scores were reported for disease-related worries and social function.

## Discussion

[^177^Lu]Lu-DOTA-TATE is indicated for the treatment of unresectable or metastatic, progressive, well-differentiated (G1 and G2), SSTR-positive GEP-NETs in adults. [13, 14]. Acute AEs were limited and were usually mild, predictable, and self-limiting; severe long-term AEs were rare [10, 14, 23, 24]. Improving patient QoL is an important consideration when evaluating the risk/benefit profiles of cancer treatments; [^177^Lu]Lu-DOTA-TATE also provides sustained and clinically significant HRQoL benefits [17].

REAL-LU aims to evaluate the real-world effectiveness of [^177^Lu]Lu-DOTA-TATE and describe the routine management and care of patients with GEP-NETs in Italy. Additionally, data on treatment safety and impact on HRQoL will be collected, and correlations between possible prognostic factors and clinical effectiveness outcomes will be explored. REAL-LU findings will help guide clinical practice by informing treatment choices and improving the selection of patients who will benefit most from PRRT.

The preliminary data reported here show that patients receiving [^177^Lu]Lu-DOTA-TATE in Italy are broadly similar in age, tumour stage at diagnosis, disease characteristics and prior procedures/treatments received to those described previously [13, 15]. The primary tumour site was more evenly balanced in the current study than in the pivotal NETTER-1 trial, which only included intestinal tumours [13]. In the current study, the primary tumour site was the ileum in 43.5% of patients, compared with 74.0% in the NETTER-1 trial. As with other studies, [^177^Lu]Lu-DOTA-TATE was primarily administered as a second-line treatment after disease progression with SSAs.

One difference between the REAL-LU study population and patients in the NETTER-1 study was that our real-world population included a high proportion of patients (71%) with grade 2 (Ki-67: 3–20%) GEP-NETs whereas the majority of the NETTER-1 trial population (66%) had grade 1 NETs [13]. With regard to the tumor grade profile, our study cohort appears to be closer closer to that of the NETTER-2 study population, which included patients with G2 or G3 advanced GEP-NETs (i.e. Ki-67 ≥ 10% and ≤ 55%). Preliminary data from NETTER-2 indicate that 35.0% of patients in that study had G3 tumours [18]. These differences between our real-world patients and those in the phase III randomised controlled trials can be further investigated when the outcomes data are available and we can investigate relationships between baseline tumour status and outcomes.

The study’s main limitations relate to its observational nature, which inherently has the potential for patient selection bias, incomplete or missing data, lack of a control group, difficulty in interpreting or verifying documented information, and variability between patients in documentation quality. The study encountered additional challenges in conducting clinical research during the COVID-19 pandemic, which impacted healthcare resources worldwide and, of particular relevance to this study, the work of nuclear medicine departments [25]. However, data obtained from non-interventional studies provide real-world information within a typical clinical setting which is more representative of the study population of interest and the clinical outcomes under observation. These factors will contribute to the relevance of the study findings when data from further analyses are available.

Despite the promising results seen with PRRT, the histological heterogeneity and high variability of SSTR2 expression in well-differentiated GEP-NETs makes it important to identify patients most likely to benefit from [^177^Lu]Lu-DOTA-TATE therapy [26]. Identifying reproducible, easily accessible, reliable, and cost-effective predictive biomarkers is crucial to optimise and improve treatment outcomes with [^177^Lu]Lu-DOTA-TATE. By exploring the correlations between potential prognostic biomarkers and clinical effectiveness outcomes, REAL-LU may identify more sensitive and reliable biomarkers for patient selection for PRRT.

## Conclusions

The REAL-LU study was designed to characterise real-world prescribing habits and clinically significant endpoints for [^177^Lu]Lu-DOTA-TATE treatment in Italy. The results of this pre-specified interim analysis found that patients receiving [^177^Lu]Lu-DOTA-TATE for GEP-NETs in Italy are broadly similar to NETTER 1trial population, but with a higher proportion of patients with a grade 2 (71%). With regard to the tumor grade profile, our study cohort appears to be closer to that of NETTER 2 study population, which included patients with G2 or G3 advanced GEP_NETs (i.g. Ki-67 ≥ 10% and ≤ 55%). Further analysis, including safety, effectiveness, survival data, and the impact of [^177^Lu]Lu-DOTA-TATE on HRQoL and healthcare resource utilisation from an Italian perspective, can be anticipated as planned study milestones are reached.

### Supplementary Information

Below is the link to the electronic supplementary material.

Supplementary file1 (DOCX 23 KB)

## Data Availability

The datasets generated during and/or analysed during the current study are available from the corresponding author on reasonable request.
