# Supplementary material for: Lutetium [177Lu]-DOTA-TATE in gastroenteropancreatic-neuroendocrine tumours: rationale, design and baseline characteristics of the Italian prospective observational (REAL-LU) study
Source: Eur J Nucl Med Mol Imaging. 2024 May 22;51(11):3417–27. doi: 10.1007/s00259-024-06725-7 (PMC11368969; doi:10.1007/s00259-024-06725-7)
Supplement: Supplementary file 1 — Supplementary file1 (DOCX 23 KB) [file 259_2024_6725_MOESM1_ESM.docx]

**Lutetium (^177^Lu)-DOTA-TATE in gastroenteropancreatic-neuroendocrine tumours: rationale, design and baseline characteristics of the Italian prospective observational (REAL-LU) study**

***European Journal of Nuclear Medicine and Molecular Imaging***

**Supplementary Material**

**Authors:** Secondo Lastoria, Marcello Rodari, Maddalena Sansovini, Sergio Baldari, Antonio D'Agostini, Anna Rita Cervino, Angelina Filice, Matteo Salgarello, Germano Perotti, Alberto Nieri, Davide Campana, Riccardo Emanuele Pellerito, Elena Pomposelli, Valeria Gaudieri, Giovanni Storto, Chiara Grana, Alberto Signore, Giuseppe Boni, Francesco Dondi, Gabriele Simontacchi and Ettore Seregni

**Corresponding author:** Sergio Baldari

UOC Medicina Nucleare, AOU Policlinico G. Martino, Via Consolare Valeria, 1 - 98125 Messina, Italy

**Email:** sbaldari@unime.it

**Supplementary Methods**

**Imaging**

Guidelines recommend that imaging (computed tomography [CT] or magnetic resonance imaging) is carried out every 6 months in G1 neuroendocrine tumours (NETs) or G2 NETs with low Ki-67 (<5.0%), and every 3 months in G2 NETs with Ki-67 >5.0%. Other recommendations include imaging with gallium-68–conjugated somatostatin receptor (SSTR) targeting peptide-positron emission tomography (^68^Ga-SSTR-PET) in combination with CT or, if not available, with SSTR scintigraphy as a considerably less sensitive alternative [1]. The use of positron emission tomography (PET) with [^18^F]fluorodeoxyglucose (FDG) is optional in NETs; combined SSTR imaging and FDG PET/CT is complementary for lesion detection but still suggested [2].

**Supplementary Table S1** Location of REAL-LU study sites

| Alessandria, Italy |
| --- |
| Bologna, Italy |
| Brescia, Italy |
| Cona, Italy |
| Firenze, Italy |
| Latina, Italy |
| Meldola, Italy |
| Messina, Italy |
| Milano, Italy (2 sites) |
| Napoli, Italy (2 sites) |
| Negrar, Italy (2 sites) |
| Padova, Italy |
| Pisa, Italy |
| Reggio Emilia, Italy |
| Rionero in Volture, Italy |
| Roma, Italy (2 sites) |
| Rozzano, Italy |
| Torino, Italy |

**Supplementary Table S2** Past or concomitant diseases or procedures reported at REAL-LU study entry by ≥5.0% of evaluable patients

| **MedDRA System Organ Class, n (%)** | **N = 161** |
| --- | --- |
| At least one past/concomitant disease or past surgery | 145 (90.1) |
| Cardiac disorders | 23 (14.3) |
| Endocrine disorders | 30 (18.6) |
| Gastrointestinal disorders | 51 (31.7) |
| Injury, poisoning and procedural complications | 9 (5.6) |
| Metabolism and nutrition disorders | 55 (34.2) |
| Musculoskeletal and connective tissue disorders | 14 (8.7) |
| Neoplasms benign, malignant and unspecified^a^ | 24 (14.9) |
| Nervous system disorders | 12 (7.5) |
| Psychiatric disorders | 13 (8.1) |
| Reproductive system and breast disorders | 14 (8.7) |
| Respiratory, thoracic and mediastinal disorders | 10 (6.2) |
| Surgical and medical procedures | 82 (50.9) |
| Vascular disorders | 75 (46.6) |

*MedDRA* Medical Dictionary for Regulatory Activities, version 23.0

^a^Including cysts and polyps. Each patient could report more than one medical condition

**References**

1. Pavel M, Öberg K, Falconi M, Krenning EP, Sundin A, Perren A, et al. Gastroenteropancreatic neuroendocrine neoplasms: ESMO Clinical Practice Guidelines for diagnosis, treatment and follow-up. Ann Oncol. 2020;31:844–60. <https://doi.org/10.1016/j.annonc.2020.03.304>.

2. Carideo L, Prosperi D, Panzuto F, Magi L, Pratesi MS, Rinzivillo M, et al. Role of combined [^68^Ga]Ga-DOTA-SST analogues and [^18^F]FDG PET/CT in the management of GEP-NENs: a systematic review. J Clin Med. 2019;8:1032. <https://doi.org/10.3390/jcm8071032>.
